# Relationship between cell stiffness and stress fiber amount, assessed by simultaneous atomic force microscopy and live-cell fluorescence imaging

**DOI:** 10.1007/s10237-015-0706-9

**Published:** 2015-07-24

**Authors:** Núria Gavara, Richard S. Chadwick

**Affiliations:** School of Engineering and Materials Science, Queen Mary University of London, Mile End Road, London, E1 3NS UK; Auditory Mechanics Section, National Institute on Deafness and Other Communication Disorders, National Institutes of Health, Building 10 Rm. 5D49, 10 Center Drive, MSC-1417, Bethesda, MD 20892 USA

**Keywords:** Cytoskeleton, Stress fibers, Actomyosin, Atomic force microscopy, Cell mechanics, Image processing and quantification

## Abstract

Actomyosin stress fibers, one of the main components of the cell’s cytoskeleton, provide mechanical stability to adherent cells by applying and transmitting tensile forces onto the extracellular matrix (ECM) at the sites of cell–ECM adhesion. While it is widely accepted that changes in spatial and temporal distribution of stress fibers affect the cell’s mechanical properties, there is no quantitative knowledge on how stress fiber amount and organization directly modulate cell stiffness. We address this key open question by combining atomic force microscopy with simultaneous fluorescence imaging of living cells, and combine for the first time reliable quantitative parameters obtained from both techniques. We show that the amount of myosin and (to a lesser extent) actin assembled in stress fibers directly modulates cell stiffness in adherent mouse fibroblasts (NIH3T3). In addition, the spatial distribution of stress fibers has a second-order modulatory effect. In particular, the presence of either fibers located in the cell periphery, aligned fibers or thicker fibers gives rise to reinforced cell stiffness. Our results provide basic and significant information that will help design optimal protocols to regulate the mechanical properties of adherent cells via pharmacological interventions that alter stress fiber assembly or via micropatterning techniques that restrict stress fiber spatial organization.

**Electronic supplementary material:**

The online version of this article (doi:10.1007/s10237-015-0706-9) contains supplementary material, which is available to authorized users.

## Introduction

A variety of cellular functions such as cell migration, proliferation, differentiation or metabolic activity require an exquisite dynamical tuning of the cell’s mechanical properties (Levental et al. 2009; Fu et al. 2010; Dupont et al. 2011). In most eukaryotic cells, mechanical stability is provided by its cytoskeleton (CSK), which is a hierarchical meshwork of polymeric proteins. The CSK is also actively involved in the application of forces onto cell–cell and cell–extracellular matrix (ECM) adhesions, thus having a crucial role in a plethora of cellular functions (Rodriguez et al. 2003; Parsons et al. 2010). Actin filaments, intermediate filaments and microtubules are the three major components of the cytoskeleton (Fletcher and Mullins 2010). Among them, actomyosin stress fibers are believed to have the most significant contribution to the modulation of cell’s stiffness and internal tension. On the basis of their subcellular location and molecular composition, stress fibers have been divided into three classes: ventral, dorsal and transverse arcs (Naumanen et al. 2008). At the microscopic level, stress fibers are composed of antiparallel arrays of F-actin bundles (approximately 10–30 actin filaments) stabilized by actin-binding proteins and interleaved with the molecular motor non-muscle myosin II (Thoresen et al. 2011; Chang and Kumar 2013).

The contribution of stress fibers to cell mechanics and morphology has been studied both in vivo and in vitro to a great extent (Howard and Hyman 2003; Herrmann et al. 2007; Lu et al. 2008; Stricker et al. 2010; Thoresen et al. 2011, 2013). In vitro studies have focused on minimal cross-linked actin networks, usually varying the relative concentrations of actin to cross-linkers and/or myosin (Howard and Hyman 2003; Thoresen et al. 2011). Those studies have been accompanied by modeling approaches aimed at predicting how mechanical parameters such as Young’s modulus (*E*) or storage modulus $(G^{'})$ increase with increasing concentrations of actin or cross-linkers. A variety of concentration $(\rho )$ dependences have been predicted depending on the assumed nature of the networks and the probing frequency regime, and they range from $\rho ^{1}$ to $\rho ^{5/2}$ [see Chapter 6 in Boal (2012) for a detailed review]. Nevertheless, these approaches are based on building and modeling homogeneous isotropic networks, dismissing both the microscopic organization of stress fibers in antiparallel bundles of F-actin, as well as the exquisite macroscopic localization of stress fibers within cells.

On the other hand, in vivo studies to evaluate the relationship between cytoskeletal organization and cell mechanics have been partly qualitative. A typical approach has been to perform dosage–response studies, using pharmacological agents known to enhance or inhibit cytoskeletal assembly, to then quantify their effect on cell mechanics and cell shape (Wakatsuki et al. 2001; Schulze et al. 2009). Nevertheless, most studies simply assess cytoskeletal reorganization in a qualitative way (Titushkin and Cho 2007; Pogoda et al. 2012; Prabhune et al. 2012). Even though researchers can now readily measure many-fold changes in the mechanical properties of individual cells, they cannot relate them to actual changes in the amount of stress fibers present in said cells (Haga et al. 2000; Rotsch and Radmacher 2000; Kuznetsova et al. 2007; Vargas-Pinto et al. 2013). Therefore, a robust quantitative correlation between the amount of actin or myosin assembled in stress fibers and cell stiffness is currently missing. In addition, there is even less understanding on how cytoskeletal architecture (i.e., thickness and global alignment of fibers) modulates cell stiffness. Such combined information is essential to bridge the gap between in vitro and in vivo approaches and to enable further studies aimed at altering cell function through the modulation of actomyosin-based tension using pharmacological interventions or substrate micropatterning.

Correlation of cell mechanics and cytoskeletal composition requires simultaneous measurement of cell stiffness and quantitative imaging of the cell’s cytoskeleton, preferentially at the single-cell level and in living cell conditions. Of the many techniques developed to measure mechanical properties in living cells, atomic force microscopy (AFM) is particularly advantageous, since it allows measurement of global or local mechanical properties in physiological conditions and with good spatial and temporal resolution (Kuznetsova et al. 2007; Raman et al. 2011). It should be noted that the majority of the studies performed to date have focused on the mechanical properties of the thickest cytosolic area in the vicinity of the nucleus, due to limitations in AFM measurements arising from the stiff substrate underneath the probed cell. To overcome this constraint, we have recently presented a new analytical model for AFM nanomechanics measurements, which allows us to reliably probe the whole cell surface using larger indentations (Gavara and Chadwick 2012). By doing so, we can now measure the global contribution of actomyosin stress fibers to cell stiffness.

We have combined our AFM-based nanomechanics approach with simultaneous fluorescence imaging of cells. Probed cells were transfected with plasmids driving the expression of GFP bound to cytoskeletal proteins of interest (actin, myosin IIa and tubulin). The use of GFP tagging, rather than immunostaining, as used in Kidoaki et al. (2006), allows us for the first time to simultaneously probe and image the same living cell, thus obtaining an accurate quantitative relationship between cell stiffness and CSK assembly. Taking advantage of the filamentous nature of the CSK, we have devised an approach to easily identify and quantify linear polymeric proteins from epifluorescence microscopy images in a single-cell basis. Furthermore, we also characterize the architecture of the CSK, by assessing the alignment of fibers (or its lack thereof), the apparent fiber thickness and whether stress fibers are preferentially located at the cell center or periphery. Using this approach, we begin to untangle the relative contribution of stress fiber amount and architecture to the mechanical properties of living cells.

Our combined AFM–GFP imaging approach yields a strong correlation between actomyosin fiber amount and stiffness of the cytoskeleton. Interestingly, changes in the amount of myosin in stress fibers have a stronger effect on cell mechanics than those associated with actin. Furthermore, the organization of the CSK has a weaker effect on cell stiffness, with aligned fibers, thicker fibers or increased density of fibers located in the cell periphery giving rise to reinforced cytoskeletons. Conversely, cell spread area or microtubule assembly had no marked effect on cell stiffness. The results presented in this study will be useful to design optimal strategies to modulate internal cell tension by pharmacological means (favoring drugs which regulate myosin activity) or by substrate micropatterning (favoring changes in the aspect ratio rather than total spread area of the patterned surfaces).

## Methods

### Cell culture and transfection

Measurements were taken in murine fibroblasts, cell line NIH3T3 (CCL-1658, ATCC). The culture medium consisted of HEPES-buffered DMEM (Gibco) with 10 % calf serum (SAFC Biosciences) and 1:100 penicillin–streptomycin (Sigma). Cells were routinely passaged in tissue culture flasks, but they were transferred to glass-bottomed petri dishes (Corning) coated with fibronectin (Sigma) to carry out the experiments. Cells were allowed to grow in glass-bottomed petri dishes for at least 2 days, but cell media were changed to serum-free media the evening before experiments to inhibit cell migration. Cells were transfected using Lipofectamine LTX (Life Technologies) following the recommended protocol for NIH3T3 cells. The following plasmids were used: pEGFP-Actin (Clonetech), Myosin-IIA-GFP (Addgene, plasmid #38297) and EGFP-Tubulin (Addgene, plasmid #12298). For immunostaining experiments, cells were fixed with 3.7 % paraformaldehyde for 30 min, permeabilized with 0.1 % Triton-X for 5 min and stained with TRITC-Phalloidin (Sigma) at 1:1000 dilution.

### Experimental setup

Measurements were taken on a Catalyst AFM (Bruker Corp.) instrument mounted on the stage of an Axiovert 200 inverted epifluorescence microscope (Zeiss) placed on a vibration isolation table (Isostation). Fluorescence images of the cells were acquired with a cooled CCD camera (Orca R2, Hamamatsu). V-shaped gold-coated silicon nitride cantilevers with four-sided pyramidal tips (MLCT, Bruker Corp.) were used as probes. Nominal spring constant of the cantilevers was $0.1\hbox { Nm}^{-1}$, but the actual spring constant of each cantilever was measured before starting the experiments using the thermal fluctuations method.

### Experimental protocol

At the beginning of the experiment, the petri dish was placed on the stage of the microscope and the cantilever was positioned far above the glass surface and allowed to thermally equilibrate. Then, the relationship between photodiode signal and cantilever deflection was calibrated by taking a force–displacement curve at a bare region of the glass and measuring its slope. We then looked for an adhered cell exhibiting suitable levels of GFP plasmid and imaged it using 20$\times$ magnification and epifluorescence conditions. Imaging parameters (exposure time and gain) were kept constant for all experiments. Immediately after imaging, the AFM contact mode was engaged and cell mechanical probing took place. We used the point-and-shoot feature to perform line scans (300 data points) across the cell. The trigger mode was set to ‘relative.’ In this option, the feedback system readjusts the initial piezo-position for each force–displacement ramp so that the maximal force applied to the cell remains constant. Line scans always started over a bare glass location close to the gel periphery. The recorded initial piezo-position at that location was used later on as a zero-height reference when computing the cell height at each location. We used $5\hbox {-}\upmu \hbox {m}$ ramps with up to $\sim$1500 nm indentations at 1 Hz. The combined process of imaging and probing required $<$8 min. Petri dishes remained on the stage of the microscope for a maximum of 1 h, allowing $\sim$6 cells to be probed for each petri dish.

### Quantification of fiber amount and alignment from fluorescence images

The algorithm for quantification of fiber amount is written in MATLAB (the MathWorks), and it is based on three independent steps: (1) initial image segmentation, (2) fiber refinement and (3) determination and subtraction of background.

**Fig. 1:**
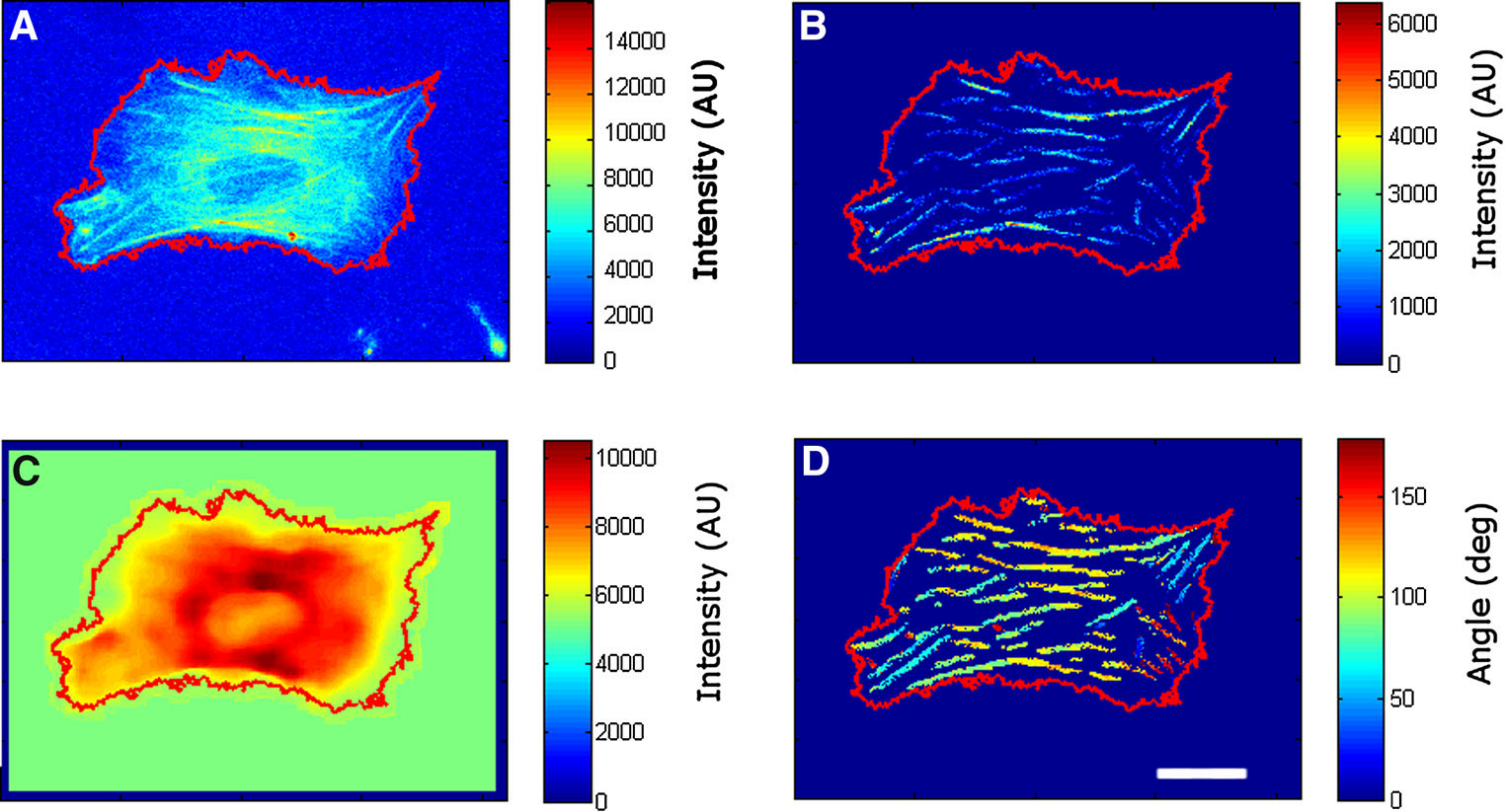
Mapping fiber position, brightness and local orientation in living cells. The panels depict a representative NIH3T3 cell transfected with GFP-actin. Raw image (*top left*), fluorescence intensity of segmented fibers (*top right*), fluorescence intensity of unbound GFP-actin (*bottom left*) and local orientation of segmented fibers (*bottom right*). *Color scale* indicates fluorescence intensity (*top and bottom left panes*) or angular direction (*bottom right)*. For angular direction, *dark blue* indicates direction toward the *bottom* edge of the paper ($0^{\circ }$), *red* indicates the direction toward the *top* edge of the paper ($179.9^{\circ }$), and *yellow-green* indicates *left–right* direction ($90^{\circ }$). All angular directions represent projections within the plane encompassed by the paper. *Scale bar* is $25~\upmu \hbox {m}$

Initial segmentation is performed as described in Zemel et al. (2010), using the convolution of the original image with a series of elongated Laplace of Gaussian (eLoG) kernels initially described by Haralick and Shapiro (1992). We used rotation steps of $\uppi /30$, thus giving rise to $n=30$ response images. Once the maximum response image is obtained and thresholded, the resulting binary image specifies the pixels corresponding to fibers. The second output of this step is a map of the local orientation of the fibers (LOF) (Fig. 1d), which is obtained by selecting, for each pixel, the rotation angle of the eLoG kernel that yielded the highest response value. Unlike (Zemel et al. 2010), we do not add any subtraction of bright spots in our algorithm, because those artifacts are corrected in the next step of the image processing algorithm.

Fiber refinement is carried out using coherence-enhancing diffusion filtering (CEDF), which is particularly suited for the completion of interrupted lines or the enhancement of flow-like structures (Weickert 1999). This algorithm, which was initially proposed by Weickert, has been incorporated into the image edge enhancing coherence filter toolbox developed by Kroon and Slump (2009). The binary image corresponding to the location of the fibers is first enhanced using the CEDF algorithm, to expand and connect interrupted fibers. Then, the local orientation of each pixel corresponding to a fiber is compared to the orientation of all the other pixels within a [9 $\times$ 9] neighborhood that also belong to a fiber, using the LOF map obtained in step 1. Only pixels whose orientation $(\theta _{\mathrm{p}})$ is very similar to its neighbors $(\theta _{\mathrm{n}})$, measured as ${<}\mathrm{cos}(\theta _{\mathrm{p}}-\theta _{\mathrm{n}})>> 0.995$, are accepted. These two steps (fiber enhancement followed by fiber trimming) are iterated until no changes take place in the binary fiber map. The fiber refinement step allows us to make sure interrupted lines are connected and also removes the effect of bright dots without the need to artificially removing elements from the resulting fiber image.

The last step involves the determination of the background GFP fluorescence, which is due to the presence of unbound monomers of GFP protein. It should be noted that background fluorescence in our computations is different than the more typical background noise due to non-optimal imaging conditions or uneven illumination. Rather, our GFP fluorescence values tend to correlate with the thickness of the cell at each pixel location and are affected by the presence of elements, such as the nucleus, that exclude GFP protein. To obtain the unbound protein map (Fig. 1c), we used [21 $\times$ 21] filtering windows and computed for each window the median value of the fluorescence intensity of the original image. Nevertheless, pixels belonging to a fiber were not used when computing the median of the window. The result of this step is a smoothly changing intensity map with no apparent fibers, likely resembling the thickness profile of the cell. Lastly, the unbound protein map is subtracted from the original image, and only pixels that have positive intensity values and belong to a fiber are accepted to generate an F-protein map (Fig. 1b). The sum of the F-protein map is then used as a measure of the amount of GFP protein in filamentous form $(F_\mathrm{GFP})$.

**Fig. 2:**
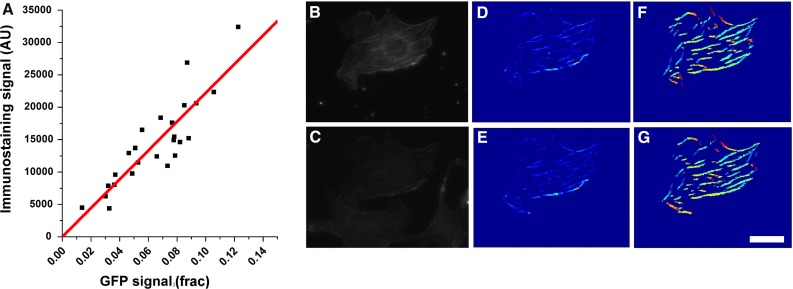
Quantification of fiber amount yields equivalent results for immunostaining or GFP-based protein tagging. Plot shows results for cells transfected with GFP-actin and subsequently immunostained with TRITC-Phalloidin. Values for immunostaining correspond to the total intensity of the segmented fibers as determined by the image analysis code. Values for GFP were analyzed similarly to first compute $F_\mathrm{GFP}$ and were then rescaled using $P_\mathrm{endo}$ as described in *Materials and Methods* section. Each data point corresponds to one cell. Images **b**–**g** are for an example cell, where the *top* row shows image processing carried out using the image obtained on the GFP channel, whereas the *bottom* row corresponds to the results for the TRITC channel. Shown are raw images (**b** and **c**), fluorescence intensity of segmented fibers (**d** and **e**) and local orientation of fibers (**f** and **g**). *Scale bar* is $25~\upmu \hbox {m}$

### Calibration of total filamentous protein

Image quantification can be used directly to measure the total amount of protein in filamentous form when cells are stained with the appropriate antibodies, and staining and imaging conditions are kept constant throughout the measurements. Nevertheless, when GFP tagging is used, the fluorescence intensity of the fibers depends directly on the expression levels of exogenous GFP protein, which are different for every cell. The amount of exogenous GFP protein $(P_\mathrm{GFP})$ can be measured as the total fluorescence intensity of the cell and then used to rescale $F_{\mathrm{GFP}}$ to obtain the total amount of protein in filamentous form $(F_\mathrm{total})$. To do so, we have assumed that the probability of GFP monomers binding to a fiber is the same as the probability of endogenous monomers:

1 $$\begin{aligned} \frac{F_\mathrm{endo}}{P_\mathrm{endo}}=\frac{F_\mathrm{GFP}}{P_\mathrm{GFP}}= \frac{F_\mathrm{total}}{P_\mathrm{total}} \end{aligned}$$

where the endo subscript refers to endogenous protein and total refers to the total amount of protein, as:

2 $$\begin{aligned} P_\mathrm{total} =P_\mathrm{endo} +P_\mathrm{GFP} \end{aligned}$$

Combining these two simple equations, we obtain:

3 $$\begin{aligned} F_\mathrm{total} =F_\mathrm{GFP} \left( {1+\frac{P_\mathrm{endo}}{P_\mathrm{GFP}}} \right) \end{aligned}$$

where $P_\mathrm{endo}$ ends up being a scaling factor which is unknown. To determine it, we have fixed and stained subsets of cells which had been previously transfected, following the same transfection and culture protocols as those used for the AFM experiments. Comparing the $F_\mathrm{total}$ values obtained from fluorescence images acquired using the GFP and the dye signal on the same cells, we were able to determine the scaling factor $P_\mathrm{endo}$ as the value which resulted in a linear fit passing along the origin, as shown in Fig. 2. It should be noted that this scaling factor is only valid for our set of measurements and our transfection protocol and should be remeasured when using a different cell line, transfection protocol or measurement condition. Finally, to enable comparisons, we present our data as the fraction of protein in filamentous form, computed as:

4 $$\begin{aligned} \frac{F_\mathrm{total}}{P_\mathrm{total}}=\frac{F_\mathrm{total}}{P_\mathrm{endo} +P_\mathrm{GFP}} \end{aligned}$$

### Computation of parameters describing cytoskeletal organization

To measure apparent fiber thickness (FT), we first compute the average value of the pixel intensities corresponding to fibers in the F-protein map. Nevertheless, this average value corresponds only to the amount of GFP-tagged protein $(\hbox {FT}_\mathrm{GFP})$. Similar to the method used to compute the total amount of protein in filamentous form, $\mathrm FT_\mathrm{GFP}$ is scaled using Eq. 3 above to obtain FT. Fiber alignment (FA) and average fiber orientation $(\bar{\theta })$ are assessed by computing the circular variance and circular mean of the values obtained in the LOF map as (Fisher 1993):

5 $$\begin{aligned}&\hbox {FA}=1-\sqrt{\bar{C}^{2}+\bar{S}^{2}} \end{aligned}$$

6 $$\begin{aligned}&\bar{\theta } =\mathrm{ArcTan}\left( {\frac{\bar{S}}{\bar{C}}}\right) \end{aligned}$$

where

7 $$\begin{aligned} \bar{C} =\frac{1}{N}\sum \limits _{n=1}^{N}\cos \theta _{n} \end{aligned}$$

and

8 $$\begin{aligned} \bar{S} =\frac{1}{N}\sum \limits _{n=1}^{N}\sin \theta _{n} \end{aligned}$$

and $\uptheta _{\mathrm{n}}$ corresponds to the local orientation of each pixel belonging to a fiber, as computed in Sect. 2.4 above. FA values close to 0 will indicate the presence of very aligned fibers, whereas a value close to 0.5 indicates randomly oriented fibers. Finally, we also assess whether fibers are preferentially located at the cell center or periphery. To do so, the projected area of the cell is first successively eroded, giving rise to 1-pixel-thick concentric rings. Then, the average pixel intensity within said rings is computed (using the F-protein map), to obtain a radial profile of fiber density. It should be noted that the radial axis is normalized so that the outermost rings correspond to radial values close to 1, whereas the innermost rings have radial values close to 0. Finally, radial location (RL) is computed as the radial position with a largest value for fiber density.

### Analysis of force–indentation curves

All computations were performed using MATLAB (the MathWorks). For each force–displacement $(d-Z)$ curve, Young’s modulus (*E*) was estimated with nonlinear least-squared fits using our bottom effect cone correction (BECC) (Gavara and Chadwick 2012):

9 $$\begin{aligned} F= & {} \frac{8E\tan \theta \delta ^{2}}{3\pi }\nonumber \\&\times \left\{ {1+1.7795\frac{2\tan \theta }{\pi ^{2}}\frac{\delta }{h}+16\left( {1.7795} \right) ^{2}\hbox {tan}^{2}\theta \frac{\delta ^{2}}{h^{2}}}\right\} \nonumber \\ \end{aligned}$$

in which *F* is the applied force, $\delta$ is indentation, $\upalpha$ is the half-opening angle of the cone, and Poisson’s ratio is assumed to be 0.5. The applied force can be expressed in terms of the deflection of the cantilever (*d*) and the spring constant of the cantilever (*k*) as:

10 $$\begin{aligned} F=kd \end{aligned}$$

The indentation can be expressed as:

11 $$\begin{aligned} \delta =\left( {Z-Z_\mathrm{CP}}\right) -d \end{aligned}$$

where *Z* is the displacement of the piezo and $Z_\mathrm{CP}$ represents the piezo-position at the contact point. The contact point is identified using a sequential search algorithm as the point that maximizes the goodness of the fit $(r^{2})$ to the contact part of the indentation curve using Eq. 9.

Finally, sample height can be computed as:

12 $$\begin{aligned} h=Z_\mathrm{CP} -Z_\mathrm{glass} \end{aligned}$$

where $Z_\mathrm{glass}$ is the piezo-position at the contact point obtained on a region of bare glass.

Only force–displacement curves whose fits yielded $r^{2}>0.75$ were used for the averages.

### Data pooling and statistics

Computed *E* values for cell locations with height ${<}4~\upmu \hbox {m}$ were pooled as ‘cytoskeleton,’ whereas *E* values from locations with height larger than $5~\upmu \hbox {m}$ were pooled as ‘nuclear region.’ A final *E* value for each cell (for cytoskeleton and/or nuclear region) was obtained computing the median of all pooled values. To assess the relationship between fiber amount and CSK (or nuclear region) stiffness, values obtained for several cells were pooled together, to reduce variability. Six relationships between fiber amount and stiffness were obtained (actin, myosin or tubulin, for both CSK or nuclear region). Therefore, once fits were obtained, analysis of covariance (Scheffé’s method) was performed using MATLAB to assess which fits were significantly different from a constant model. To assess which parameters describing CSK organization (FA, FT or RL) had a significant effect on CSK reinforcement, we performed F-tests to compare linear models containing different combinations of parameters. Throughout the manuscript, errors are indicated as SE and *p* values reported for fits to data indicate probability versus constant model.

## Results

### Imaging and quantification of GFP-transfected cells

The transfection protocol we used yielded $\sim$24 % transfected cells, with large variability in their total fluorescence intensity. Transfected cells displayed no marked morphological differences with those not transfected, with the exception of cells expressing very high levels of GFP protein. Those cells (which were not used for our experiments) were markedly brighter, had much larger spread areas than other transfected cells and were usually multinucleated. We also discarded cells which were very dim, because we could not correctly visualize or extract their fibers using our analysis algorithm. On average, cells used in our experiments contained $\sim$12 % exogenous GFP protein, and higher levels of exogenous GFP protein did not translate into impaired fiber assembly (Suppl Fig 1). This result confirms that cells were able to assemble actomyosin fibers and tune the composition of their CSK in spite of having extra protein containing a GFP tag. Together, these results indicate that we can readily use GFP as reporter to quantify CSK organization and composition in living cells without interfering with filament assembly or cell morphology.

Imaged cells displayed marked fibers, many of them running in parallel, in distinct families (Figs. 1, 2). Using our algorithm, we dissected out fibers from the background intensity arising from the monomeric GFP-tagged protein and quantified both fiber pixel intensity (FPI) and local orientation of fibers (LOF). It should be noted that fibers located over thicker areas of the cell appeared artifactually brighter in the raw images. This phenomenon was accounted for and corrected in our image processing analysis, which yielded fibers which had constant intensity levels along their length. Intensity maps of background fluorescence levels showed a central disk with the lowest fluorescence levels (likely to correspond to the nucleus) surrounded by a region displaying the highest background intensities (likely corresponding to the thick cytosolic areas in the vicinity of the nucleus). Such background distribution for unbound GFP protein is expected, since somatic cell nuclei contain much lower levels of actin than the cytoplasm (Stüven et al. 2003) as well as exhibit low permeability to standard GFP-actin and other fluorescent probes (Belin et al. 2013). The levels of background intensity in the nucleus area were similar to the background intensities at the furthermost part of the cell periphery, likely indicating that the cytoplasm found immediately above or below the nucleus was very thin.

### Morphological and cytoskeletal cellular variability

Previous studies have used substrate micropatterning to modulate cell morphology and cytoskeletal composition (Roca-Cusachs et al. 2008; Park et al. 2010). These approaches were based on restricting cell area and shape, which has a great influence in important cell functions and can even lead to apoptosis (Connelly et al. 2010; Yan et al. 2011). Conversely, we have used cells cultured at very low density in unrestricted spreading conditions, taking advantage of their inherent morphological variability. Cell areas ranged between 600 and $12000~\upmu \hbox {m}^{2}$, with aspect ratios up to 5. Maximum cell heights were measured in the vicinity of the nucleus and were in the order of 6–8 $\upmu \hbox {m}$. We did obtain CSK polymerization levels that spanned for almost 1.5 decades for both actin and myosin, although variability for cell transfected with GFP-tubulin was more limited. Similarly, our sample contained a good variety of CSK architectures, ranging from very aligned fibers to random fiber distributions. In particular, we found a strong correlation between cell aspect ratio and actin or myosin fiber alignment ($p<0.001$ for both actin and myosin), while no correlation was found in the case of microtubule networks ($p=0.08$) (Suppl Fig 2a). In addition, the direction of the fibers tended to align with the major axis of the cell, especially in cells displaying high aspect ratios (Suppl Fig 2a). Finally, our cells also displayed large spread for values of apparent fiber thickness, with a $\sim$tenfold difference between the largest and the smallest values measured. Together, these results indicate that we can obtain a good range of CSK polymerization levels, architectures and cell spreading areas only relying on inherent cell variability.

### AFM probing of GFP-transfected cells

Similar to the variability observed for fiber amount, Young’s modulus (*E*) values obtained for probed cells spanned one order of magnitude. We observed similar *E* ranges for transfected cells (with all three plasmids) and non-transfected cells (Suppl. Fig 4), indicating that the presence of the GFP tag did not markedly affect the ability of the exogenous protein to assemble into fibers and contribute to the cell’s mechanical stability. Concerning the effect of repeatedly probing the cells, very few of them displayed gross changes in morphology after being indented by the AFM tip. Furthermore, a subset of cells probed and imaged twice in a period of $<$30 min displayed only minor changes in fiber amount and Young’s modulus values ($<$15 % change) between the two sets of measurements. Finally, taking into account how tip-induced sample deformations extend through the depth of a probed material (Gavara and Chadwick 2009), the large indentations that we applied to our cells allowed us to probe large cell volumes for each indentation $({\sim }17~\upmu \hbox {m}^{3})$. Combining this with the fact that we probed cells at multiple locations and pooled together the obtained *E* values allows us to obtain a global value for cell stiffness, which can then be correlated with the global parameters for cytoskeletal assembly obtained from GFP imaging and quantification.

### Actin and myosin fiber assembly are the main determinants of cytoskeletal stiffness

The amount of actin and myosin present in stress fibers displayed large variability, with median values of 5.9 % for actin (denoting that 5.9 % of the cell’s total actin was found as F-actin and 94.1 % as G-actin) and 3.4 % for myosin. Combining these median values, we obtain an average ratio of myosin to actin of ${\sim }0.6$, which is similar to ratios of actin to myosin assembly required to give rise to contractile fibers in vitro (Thoresen et al. 2011) and in silico (Wang and Wolynes 2012). Furthermore, molar ratios of 0.2–0.5 (myosin/actin) have been measured in vivo in smooth muscle cells (Gabella 1984).

**Table 1:** Results of the correlation between fiber amount and cytoskeletal or nuclear region Young’s modulus

	$E_{0}$ (kPa)	*p*	$\alpha$ (kPa)	*p*	$r^{2}$
Actin					
Cytoskeleton	$0.37 \pm 0.08$	0.0014	$9.48 \pm 1.02$	$<0.001$	0.92
Nuclear region	$1.14 \pm 0.11$	$<$0.001	$6.52 \pm 1.50$	0.012	0.83
Myosin					
Cytoskeleton	$0.50 \pm 0.08$	$<$0.001	$14.67 \pm 1.91$	$<$0.001	0.87
Nuclear region	$0.81 \pm 0.12$	0.003	$8.74 \pm 2.71$	0.03	0.72
Tubulin					
Cytoskeleton	$0.93 \pm 0.40$	0.03	$3.97 \pm 14.62$	0.79	0.003
Nuclear region	$1.98 \pm 0.58$	0.008	$-6.03 \pm 18.81$	0.76	0.011

Analysis of covariances (Scheffé’s method) yielded significant differences between the fits for $\hbox {CSK}_{\mathrm{actin}}$ versus $\hbox {CSK}_{\mathrm{myosin}}~(p<0.001)$ and between $\hbox {CSK}_{\mathrm{actin}}$ versus $\hbox {NR}_{\mathrm{actin}}\,(p<0.001)$

**Fig. 3:**
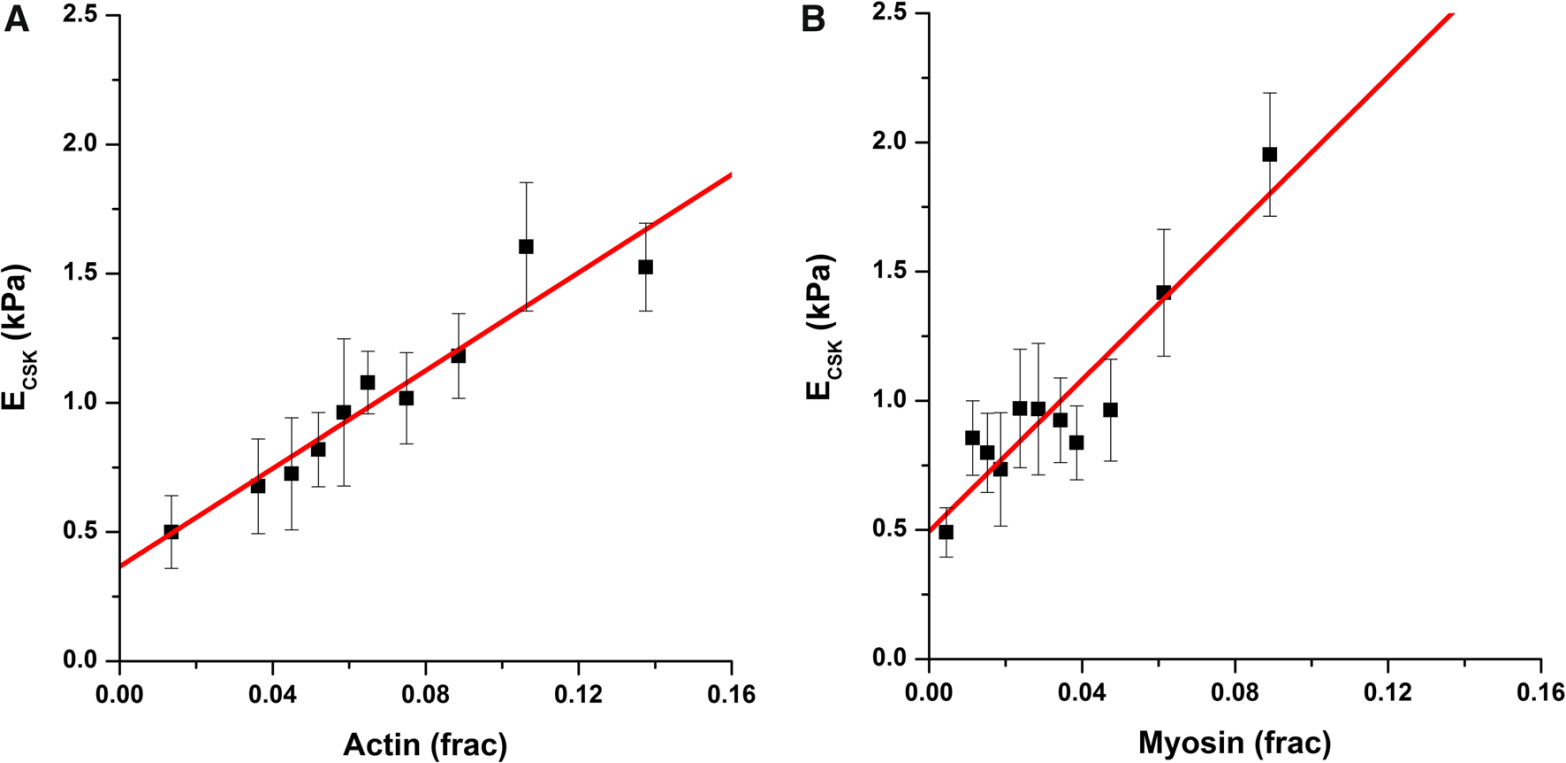
Actomyosin amount modulates cytoskeletal stiffness. Actin (**a**) and myosin (**b**) fiber amount markedly modulate cytoskeletal Young’s modulus. Fiber amount is computed as fraction. Each data point corresponds to the average of 10 cells. Fit lines correspond to a linear model

We found a strong relationship between actin and myosin fiber amount ([*F*]) and cellular *E*, which was well fitted using a linear model $(E=E_{0} + \alpha [F])$ (Table 1; Fig. 3). Analysis of covariance indicated that the fits obtained for actin and myosin were significantly different from each other $(p<0.001)$. $E_{0}$ and $\alpha$ parameters were larger for myosin data, with a significant difference versus actin values in the case of $\alpha ~(\alpha _\mathrm{ACTIN}$ vs $\alpha _\mathrm{MYOSIN}~p=0.023$; $\hbox {E0}_\mathrm{ACTIN}$ vs $\hbox {E0}_\mathrm{MYOSIN}~p=0.29$). As expected, we could not find a strong tendency between fiber amount and cell mechanics when assessing microtubules (Suppl. Fig 5) (Celik et al. 2013). $E_{0}$ values were similar to those obtained in micropipette aspiration experiments (Guilak et al. 2000; Hochmuth 2000). In those experimental conditions, cells are not attached and contain only a thin layer of actin assembled as a dendritic network, without the presence of stress fibers (Clark et al. 2013). Therefore, $E_{0}$ is likely to correspond to the stiffness of the dendritic network, whose visualization and quantification were not possible using our methodological approach.

### Cell area has no effect on cell mechanics

A large number of studies have used substrate micropatterning to modulate cell stiffness. Nevertheless, they are based on confining cells into very small islands, often altering levels of protein expression and the normal assembly of cytoskeletal proteins (Connelly et al. 2010; Yan et al. 2011). We investigated whether the same correlation between cell area and cell stiffness would persist in cells allowed to spread unconstrained. Surprisingly, we found no correlation between cell area and cell stiffness (Suppl Fig 3a, $p=0.60$), or between cell area and stress fiber assembly (Suppl Fig 3b, $p=0.83$) in those cells. Our results suggest that when the relationship between cell area and fiber amount is decoupled, so is the relationship between cell area and cell stiffness. Therefore, altering cell gross morphology appears to be insufficient to affect cell stiffness, if these modifications do not lead to actual changes in the actomyosin composition of the cytoskeleton.

### Fiber alignment and apparent thickness have a weak effect on global cell stiffness

Despite the strong correlation observed between actomyosin amount and cytoskeletal stiffness, single-cell data displayed a marked scatter around the fit line. Therefore, we next assessed whether cytoskeletal architecture (as opposed to stress fiber amount) could explain part of the observed scatter (Kidoaki et al. 2006; Kidoaki and Matsuda 2007). From our images, we could quantify fiber alignment (FA), average fiber thickness (FT) and preferred radial location (RL) of the fibers for each cell, and we considered these three parameters to be independent from each other. We defined the over (or under)-estimation of *E* versus the expected value for a particular amount of total fiber amount as $E/E_\mathrm{fit}$ and assessed whether it correlated with any of the three parameters (FA, FT or RL) using linear models such as:

13 $$\begin{aligned} \frac{E}{E_\mathrm{fit}}=a+b\frac{\hbox {FA}}{\left\langle \hbox {FA} \right\rangle }+c\frac{\hbox {FT}}{\left\langle \hbox {FT} \right\rangle }+d\frac{\hbox {RL}}{\left\langle \hbox {RL} \right\rangle } \end{aligned}$$

**Table 2:** Results of the correlation between fiber alignment, fiber thickness, radial location and cytoskeletal reinforcement

Actin		${\langle }\hbox {FA}{\rangle }$	${\langle }\hbox {FT}{\rangle }$	${\langle }\hbox {RL}{\rangle }$	
		0.22	113	0.70	

	*a*	*b*	*c*	*d*	$r^{2}$
	$1.10 \pm 0.23$	$-0.39 \pm 0.18$	–	0.45 $\pm$ 0.2	0.07
*p* values	$<$0.001	0.03	–	0.03	

Myosin		${\langle }\hbox {FA}{\rangle }$	${\langle }\hbox {FT}{\rangle }$	${\langle }\hbox {RL}{\rangle }$	
		0.19	60	0.73	

	*a*	*b*	*c*	*d*	$r^{2}$
	$0.93 \pm 0.14$	–	$0.23 \pm 0.11$	–	0.09
*p* values	$<$0.001	–	0.03	–	

Shown are only the results for the best models as assessed by *F*-tests

where the average values ${\langle }\hbox {FA}{\rangle }$, ${\langle }\hbox {FT}{\rangle }$ and ${\langle }\hbox {RL}{\rangle }$ for actin and myosin can be found in Table 2. We used F-tests to assess which combination of parameters best fitted our results for either actin or myosin. For the case of actin, the best model included the modulatory effect of fiber alignment and radial location of fibers ($p=0.034$ for F-test vs model with only 1 parameter; $p=0.026$ vs constant model). For the case of myosin, the best model included only the modulatory effect of fiber thickness ($p=0.030$ vs constant model). The signs of the parameters *b*, *c* and *d* obtained for the best fits (Tables 1, 2) indicate the cytoskeletal organizations that lead to stiffness reinforcement. As guideline, a positive value for *b* indicates a preference for randomly oriented fibers, a positive value for *c* indicates a preference for thicker fibers and a positive value for *d* indicates a preference for fibers located in the cell periphery. Using this analysis, we observed that the presence of aligned and/or peripheral actin fibers resulted in reinforced cytoskeletons (Table 2). On the contrary, when myosin was assessed, the presence of thicker fibers gave rise to higher stiffness (Table 2).

### Stress fiber amount modulates to a lesser degree the stiffness of nuclear regions

**Fig. 4:**
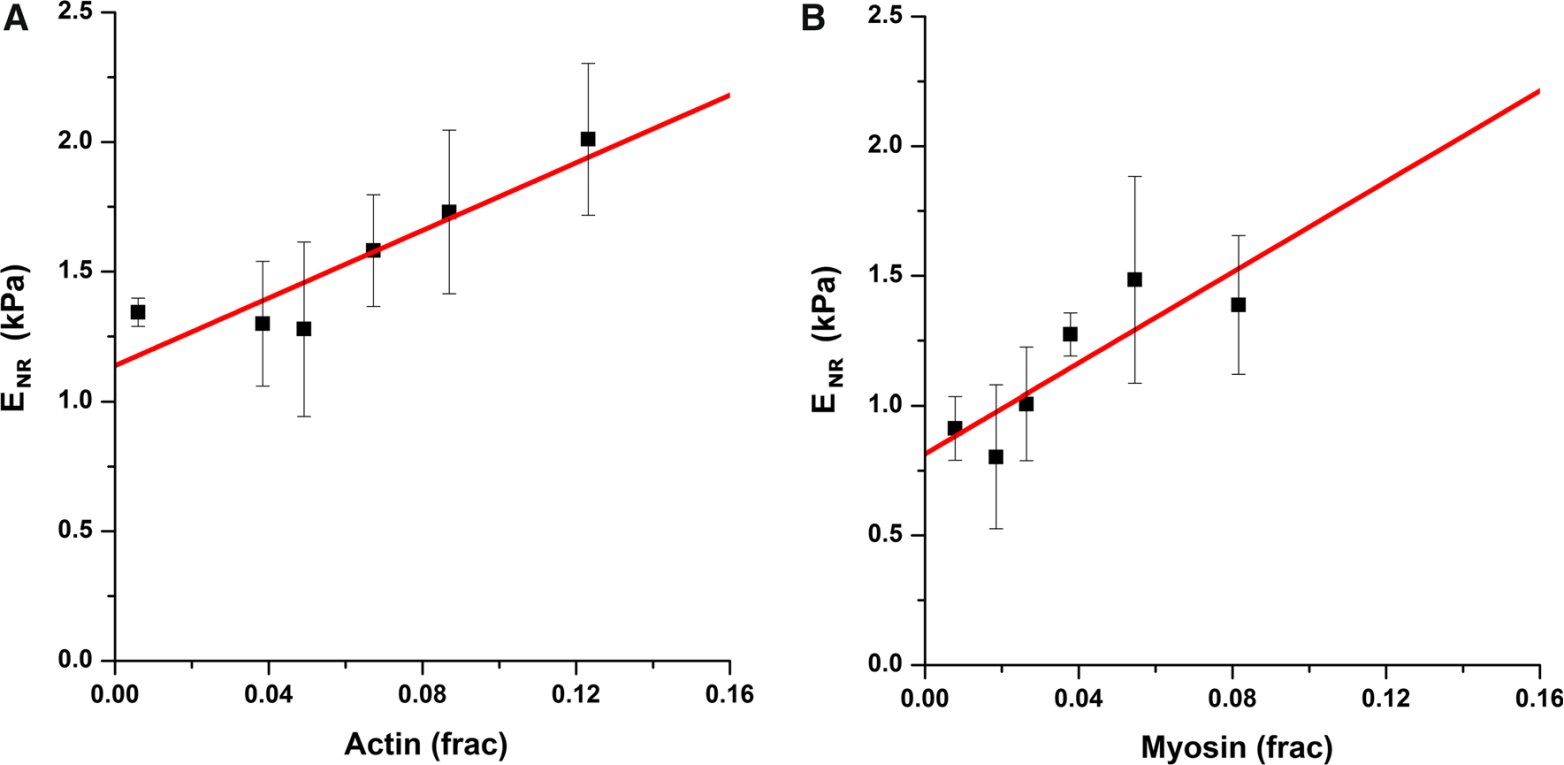
Actomyosin amount has a very mild effect on the stiffness of the nuclear area. Young’s modulus of nuclear region as a function of actin (**a**) or myosin (**b**) fiber amount. Each data point corresponds to the average of 4 cells. Fit lines correspond to a linear model

The mechanical stability of the cell nucleus is believed to arise, predominantly, from the lamin-rich inner nuclear membrane and the nuclear envelope, in a complex structure known as the nucleoskeleton (Zwerger et al. 2011). At the same time, the cell nucleus is intimately linked to the CSK via adaptor proteins known as the LINC complex (LInker of Nucleoskeleton to Cytoskeleton), and this mechanical connection is believed to mediate mechanotransduction events (Fedorchak et al. 2014). Therefore, we tested whether the strong relationship between stress fiber amount and stiffness would also hold on cellular areas near the nucleus. Similar to the results found for the cytoskeleton, we found a strong relationship between actin and myosin fiber amount ([*F*]) and cellular *E* using a linear model (Table 1; Fig. 4). Nevertheless, analysis of covariance indicated that the fits obtained for actin and myosin were not significantly different from each other ($p=0.0838$). Interestingly, $E_{0}$ values measured in nuclear regions were in the range of those previously obtained by micropipette aspiration on isolated nuclei (Guilak et al. 2000), but were lower than those obtained by AFM probing at the nanoscale using ultrasharp tips (Liu et al. 2014). This would suggest that we are probing the mechanical properties of the whole nucleus jointly with the surrounding cytoskeleton, rather than measuring only the contribution of the nuclear lamina.

## Discussion

### Limitations

In this study, we combine AFM-based nanomechanics with simultaneous fluorescence imaging of cells, followed by image quantification and analysis of fibrous structures. It should be noted that for each cell, a single fluorescence image was acquired using a 20$\times$ objective. As a result, the obtained raw images and the computed F-maps represent a 2-D projection of the true 3-D distribution of fibers. Of the parameters measured, the most affected by this projection effect is *FA*, the alignment of fibers. Taking into account that a more random orientation will result in a larger *FA* value, our imaging approach will always compute a lower bound for the alignment factor, because misalignment in the z direction will not be accounted for. Nevertheless, comparing the height profile of our cells (with cell heights up to $8~\upmu \hbox {m}$) with their spread area (average cell diameter of $\sim 70\upmu \hbox {m}$), it is expected that the angular variability in the z direction is much less than that expected in the *x*-*y* directions. Since the depth of field of our working objective is ${\sim }6\upmu \hbox {m}$, most of the fluorescence emitted by the cell will be collected by our imaging system (this includes fluorescence emitted by filamentous actin and also globular GFP-actin found in the cytoplasm). Therefore, the results obtained from our image quantification approach will likely reflect the actual amount and radial organization of stress fibers in our probed cells, while due to the 2-D projection the actual vertical location of the stress fibers (i.e., cortical or ventral) will not be assessed. Lastly, it is worth mentioning that our image analysis algorithm is based on the detection of fibrilar structures such as stress fibers. Therefore, actin or myosin assembled as a dendritic layer is not measured in this study.

Our measurements were taken in NIH3T3 fibroblasts which had been serum-deprived for a number of hours before AFM probing took place. As a result, our cells did not display a migratory phenotype, but rather exhibited stable stress fibers which remained unaltered during the course of the experiment. In addition, the cell morphology and distribution of fibers had increased symmetry, as compared to that expected for migratory cells with marked leading and trailing edges. Together, this allowed us to better assess the overall effect of cytoskeletal organization on cell stiffness. Finally, it should be noted that experiments were carried out on transformed cell line. While NIH3T3 are frequently used for AFM studies (Kidoaki et al. 2006; Kidoaki and Matsuda 2007), other studies have reported successful AFM probing using more physiologically relevant primary cell lines (Roca-Cusachs et al. 2008).

### Relationship between actin and myosin concentrations

The increase in cell stiffness with increasing amounts of actin in stress fibers was well fitted by a linear model. It should be noted that the exponent of 1 is slightly lower than the values reported in vitro for entangled isotropic and homogenous actin networks, in which a power law behavior with an exponent $>$1 has been observed when assessing the relationship between network stiffness and actin monomer concentration (Gardel et al. 2003). Similar to actin, when assessing cell stiffness the concentration dependence for myosin assembled in stress fibers also followed a linear trend. Interestingly, the slope obtained for myosin was significantly larger than the value obtained for actin (Table 1). This result highlights the different roles of actin and myosin in cytoskeletal assembly. Actin comprises the bulk of the filamentous scaffold, whereas myosin minifilaments mainly reinforce and add tension to the structure (Humphrey et al. 2002; Wagner et al. 2006; Thoresen et al. 2011). In this connection, we were also interested in the necessary interplay between the concentrations of actin and myosin that lead to stable stress fibers. For that, we used the results of the data fits to assess the concentrations of actin and myosin that give rise to the same cell stiffness. At high concentrations, we observe that the two protein concentrations grow in parallel as $[M]=0.64[A]$. Interestingly, this linear relation is not observed in actomyosin bundles reconstituted in vitro, where the concentration of myosin approximately scales as a power of two with the concentration of actin (Thoresen et al. 2011). The higher concentration of myosin required for in vitro networks may highlight the additional feature of myosin minifilaments as actin filament cross-linkers. A number of myosin proteins may be required to passively stabilize the reconstituted actomyosin bundles, whereas this role is instead performed by a plethora of ABP in living cells.

### Organization of the cytoskeleton

Our results indicate that the spatial distribution of stress fibers has a second-order modulatory effect. In particular, by assessing the ratio $E/E_\mathrm{fit}$, we have considered a certain fixed amount of protein in filamentous form (that observed for each particular cell) and attested whether it is more advantageous for cytoskeletal reinforcement to have, i.e., many thin fibers or few thicker fibers. According to our results, there are no mechanical differences associated with actin fiber thickness, which may suggest that all types of actin organization at the microscale (actin filaments, filopodia and lamellipodia) provide a similar level of mechanical support. On the contrary, myosin would be able to markedly induce cytoskeleton reinforcement simply focusing the action of minifilaments toward a reduced collection of preformed stress fibers, without the need of gross cytoskeletal reorganization.

In addition to fiber thickness, we have also assessed how the specific intracellular distribution of cytoskeletal filaments influences overall cell stiffness. In this case, we observe that the presence of aligned and/or peripheral actin fibers results in reinforced cytoskeletons, whereas the local organization of myosin fibers has no significant effect on cytoskeletal reinforcement. It is worth considering that stress fibers found in different cellular locations have different biochemical composition and origin. In particular, stress fibers located at the cell periphery (i.e., arcs) are enriched in myosin IIa, whereas central stress fibers (i.e., dorsal) incorporate myosin IIa only occasionally (Naumanen et al. 2008; Vallenius 2013). Since we have used a GFP–myosin IIa plasmid, not all myosin fiber organizations will be imaged with the same quality in our experiments. Therefore, it is expected that the effects of cytoskeletal fiber organization on cell stiffness will be more evident when using actin as a reporter of fibers. Last but not least, it is worth remembering that unconstrained cells with larger aspect ratios displayed more aligned fibers actomyosin fibers. Therefore, micropatterning approaches aimed at altering the aspect ratio of adherent cells are indeed a good method to modulate cell stiffness via changes in fiber alignment, even though the modulatory effect will not be as dramatic as changing the amount of stress fibers assembled.

### Relationship between cytoskeletal and nuclear stiffness

Previous studies comparing the stiffness of the nucleus to that of the cytoskeleton provide conflicting results. While it is believed that the nucleus is the stiffest element in the cell (Zwerger et al. 2011), some studies which depict mechanical maps of adherent cells obtained using AFM suggest otherwise (Haga et al. 2000; Kidoaki and Matsuda 2007; Park et al. 2010; Vargas-Pinto et al. 2013). It should be noted that for those AFM studies, as well as for our results, stiffness measured in the nuclear region likely reflects the mechanical contribution of both the nucleus and the surrounding cytoskeleton. In our measurements, we found cells in which peripheral regions were stiffer than nuclear regions, but also other cells in which the nuclear region was the stiffest one. This apparent inconsistency can be explained when computing the ratio $E_\mathrm{NR}/E_\mathrm{CSK}$ as a function of actin amount [*A*] using the data fits we had previously obtained (Table 1, 2). A critical point is reached when $[A]=25.89\,\%$, since $E_\mathrm{NR}$ and $E_\mathrm{CSK}$ are predicted to have the same value. As a result, for cells that have lower levels of actomyosin, the nuclear region appears stiffer than the peripheral cytoskeleton, whereas in cells that have a rich actomyosin cytoskeleton, its stiffness exceeds that of the nuclear region.

## Conclusions

In this study, we have combined for the first time quantitative measurements obtained by AFM probing and GFP-based fluorescence imaging to assess how stress fiber amount and organization modulate cellular stiffness. Our findings indicate that adherent cells can readily change their mechanical properties by multiple routes. One involves changing the total amount of stress fibers, which is governed by both actin and myosin assembly. The second one involves altering the local distribution of stress fibers and their thickness. The information obtained in this study will be useful to current efforts aimed at altering cell mechanics and function using chemical or topographical cues that affect the cell’s cytoskeleton.

## Electronic supplementary material

Below is the link to the electronic supplementary material.

**Suppl Figure 1. The presence of exogenous GFP-tagged protein does not affect cytoskeletal assembly.** Total amount of protein in filaments (computed as fraction) as a function of the level of exogenous protein (computed as *P*_*GFP*_/*P*_*endo*_). Each data point corresponds to one cell. (TIFF 21.5MB)

**Suppl Figure 2. The aspect ratio of a cell influences the orientation of its stress fibers.** (a) Fiber alignment, a value close to 0 indicates that the majority of stress fibers where aligned in the same direction whereas values close to 0.5 indicate random orientation of fibers. (b) Fiber orientation with respect to major axis of the cell, a value of 1 indicates perfect alignment of the stress fibers with the direction of the major axis of the cell. Each data point corresponds to one cell. Tubulin data are also included, even though fibrous structures observed in these cells are not stress fibers. (TIFF 177MB)

**Suppl Figure 3. Cell spreading area has no effect on cytoskeletal stiffness or fiber amount.** Cytoskeletal Young´s modulus (*A*) and fiber amount (*B*) as a function of cell area for GFP-actin (red) and GFP-myosin (blue) transfected cells. Each data point corresponds to one cell. (TIFF 168MB)

**Suppl Figure 4. Non-transfected cells displayed similar stiffness values to their transfected counterparts.** Whisker´s plot contains N=21 cells. (TIFF 21.5MB)

**Suppl Figure 5. Microtubule amount has no effect on cell stiffness.** Fiber amount is computed as fraction. Each data point corresponds to 1 cell. (TIFF 162MB)
